# Robust CRISPR/Cas9 mediated genome editing and its application in manipulating plant height in the first generation of hexaploid Ma bamboo (*Dendrocalamus latiflorus Munro*)

**DOI:** 10.1111/pbi.13320

**Published:** 2020-01-10

**Authors:** Shanwen Ye, Gang Chen, Markus V. Kohnen, Wenjia Wang, Changyang Cai, WenSha Ding, Chu Wu, Lianfeng Gu, Yushan Zheng, Xiangqing Ma, Chentao Lin, Qiang Zhu

**Affiliations:** ^1^ Basic Forestry and Proteomics Center (BFPC) Fujian Provincial Key Laboratory of Haixia Applied Plant Systems Biology College of Forestry Fujian Agriculture and Forestry University Fujian China; ^2^ Department of Molecular Cell & Developmental Biology University of California Los Angeles CA USA

**Keywords:** bamboo, genome editing, CRISPR, Cas9

Bamboo is a special grass to human due to its great economic and ecological values. Around 2.5 billion people are directly producing and consuming bamboo, and its international trade reached 68.8 billion US dollars in 2018 (Data from International Bamboo and Rattan Organization). One major bamboo species in Asia is Ma bamboo (*Dendrocalamus latiflorus Munro*), which is a hexaploid species with three subgenomes (2*n* = 72, AABBCC; Guo *et al.*, 2019). Despite its agronomic importance, it is nearly impossible to modify bamboo traits by traditional breeding as it takes over 70 years to flower. Bamboo research largely lagged behind due to the lack of efficient genetic manipulation tools.

The CRISPR (Clustered Regularly Interspaced Short Palindromic Repeat)/Cas9 provides straightforward ways for genome editing in many plants (Yin *et al.*, 2017), but has never been applied in bamboo. Here, we reported the generation of bamboo mutants with CRISPR/Cas9 technology by targeting one specific copy or all homoeologous genes.

Since our recently established genetic transformation protocol is time‐consuming (~1.5 years; Ye *et al.*, 2017), we optimized the CRISPR/Cas9 system in bamboo protoplast. We first improved the protoplast preparation methods and could isolate 3.0 x 10^6^ protoplasts/g fresh leaves. Next, we improved the PEG‐mediated transformation method and reached efficiencies of 53.3% for a single plasmid and 29.8% for two cotransformed plasmids (Figure 1A), which is sufficient for optimizing the CRISPR/Cas9 system. The maize *UBI* promoter was used to drive Cas9 expression (Ye *et al.*, 2017). Three polymerase III‐dependent promoters from rice (*OsU6a/OsU6b/OsU6c*) were selected to express the sgRNA cassettes (Ma *et al.*, 2015), as bamboo exhibits high genomic similarity with rice (Peng *et al.*, 2013). To check the effectiveness of CRISPR/Cas9 constructs, a frameshift mutated *GFP* (*mGFP*) containing an additional ‘guanine’ thereby produces no fluorescence signal was simultaneously cotransformed with CRISPR/Cas9 plasmids (Figure 1B). Around 1.8% of the protoplasts transformed with the *UBI‐Cas9/OsU6b‐sgRNA* construct showed strong signals within 72 h, indicating that the *mGFP* function was restored by the CRISPR/Cas9 system through deleting the additional ‘guanine’ (Figure 1C). The *OsU6a* and *OsU6c* promoters work as well, however, with lower efficiency than the *OsU6b* promoter, as positive signals were only occasionally observed with more than 10 replicates. Taking together, the *UBI*‐Cas9/*OsU6b*‐sgRNA construct effectively works in bamboo protoplast and was used for the following endogenous gene editing in Ma bamboo.

**Figure 1:**
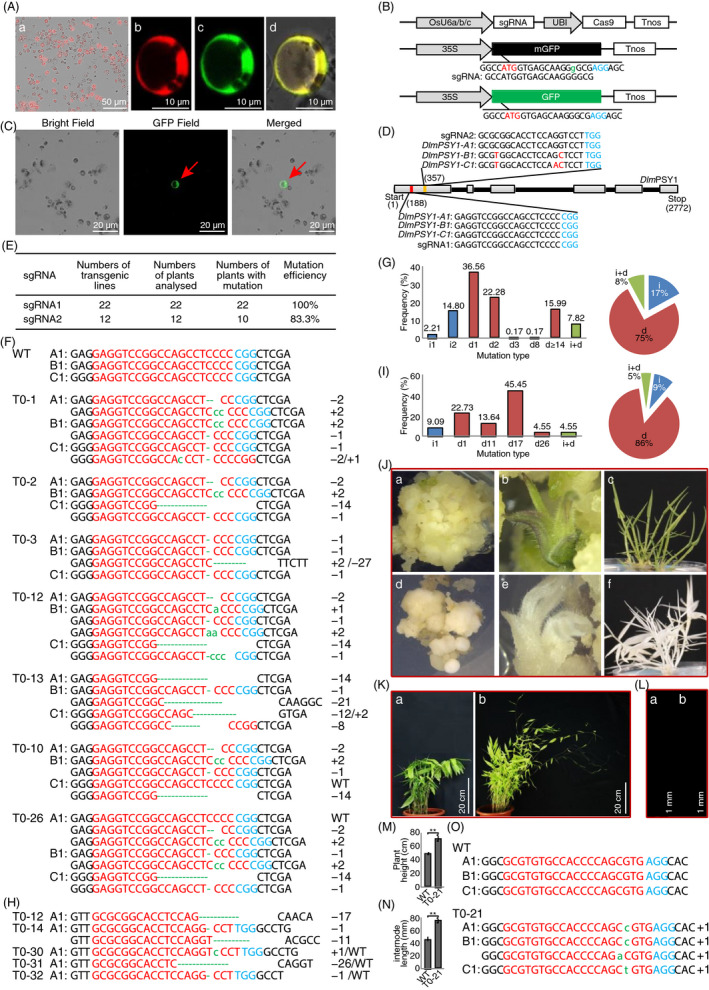
Genome editing in Ma bamboo using CRISPR/Cas9 technology (A) Bamboo protoplast isolation and transformation. a. Microscopic image of isolated bamboo protoplast transformed with *35S:tdTomato*. b‐d. Images of bamboo protoplasts co‐expressing the fluorescence proteins *tdTomato* (b) and GFP (c) driven by the 35S promoter, and their overlay (d). (B) CRISPR/Cas9 plasmids for bamboo protoplast. Top: CRISPR/Cas9 constructs expressing the sgRNA directed against *mGFP* and driven by *OsU6a*/*OsU6b*/*OsU6c,* respectively; middle: *mGFP* expression construct, *mGFP* contains one additional guanine (lower‐green case) downstream of the translational start site (red); and bottom: GFP expression construct. The sgRNA was designed to produce the presumptive cleavage site at the third nucleotide upstream of the PAM sequence (blue). (C) Representative bamboo protoplasts cotransfected with *mGFP* and *OsU6b‐sgRNA*/*UBI‐Cas9* reproducibly emitting fluorescence signals (red arrows). (D) *DlmPSY1* gene structure and sequences of the target sites. Grey boxes: exons; black lines: introns; number in brackets: positions of start codon, stop codon and sgRNA target sites (red and orange rectangles). The PAM regions (blue), SNPs (red) and nucleotide sequences of the *sgRNAs* and *DlmPSY1* genes were given. (E) Frequencies of the CRISPR/Cas9‐induced mutations in two target sites of the *DlmPSY1*. (F) Representative *DlmPSY1* mutations at the sgRNA1 site. T0‐1, T0‐2, T0‐3, T0‐12 and T0‐13 represent loss of function mutants. T0‐10 and T0‐26 lines contain heterozygote mutations in the C1 subgenome and chimeric mutations in the A1 subgenome, respectively. Red: sgRNA target regions; blue: PAM regions; green lowercase letters: nucleotide indels; dotted lines: omitted nucleotides. (G) Frequencies of indels (left) and mutation types (right) at the sgRNA1 site of *DlmPSY1.* i# and d#: # of bp inserted or deleted, respectively; d ≥ 14: more than 14 bp deletion; i + d: target sites with both deletions and insertions. (H) Representative *DlmPSY1* mutants at the sgRNA2 site. The represents homozygote (T0‐12), biallelic (T0‐14) and heterozygote (T0‐30 to T0‐32) at A1 subgenome were shown. (I) Frequencies of indels (left) and mutation types (right) at the sgRNA2 site of *DlmPSY1*. (legend: see G). (J) Phenotypes of representative *dlmpsy1* mutants. a‐c, Wild‐type; d‐f, *dlmpsy1* mutant (T0‐1). (K‐N) Phenotypes of wild‐type and the represented *grg1* mutant. Growth phenotype (K) and internode elongation (L) of 5‐month‐old wild‐type (a) and *grg1* (b) plants grown in the greenhouse. Plant heights (M) and internode lengths (N) were quantified. ∗∗: *P* < 0.01. (O) Mutations of the *GRG1* gene were confirmed by Sanger sequencing. The sgRNA target regions (red), PAM regions (blue), nucleotide insertions (green) and their length (right side) are shown.

The putative phytoene synthase (*PSY1*) in bamboo, whose homolog in maize functions in carotenoid biosynthesis (Zhu *et al.*, 2016), was selected for the initial test. Three bamboo *PSY1* alleles (*DlmPSY1‐A, DlmPSY1‐B and DlmPSY1‐C*) were identified and cloned by a homology cloning strategy (Figure 1D). To mutate all copies of *DlmPSY1*, sgRNA1 targeting a conserved site among all *DlmPSY1* loci was designed (Figure 1D). In addition, the sgRNA2 target site containing 2–3 single‐nucleotide polymorphisms (SNPs) in the spacer region among three *DlmPSY1* homoeoalleles was selected to test the tolerance of sgRNA mismatches (Figure 1D).

A total of 1600 bamboo calluses induced from stem were transformed as described previously (Ye *et al.*, 2017). In total, 34 independent transgenic lines were confirmed positive (2.1%) by PCR. Based on Sanger sequencing results, 22 (100%) and 10 (83.3%) independent T0 lines were edited in the sgRNA1 and sgRNA2 regions, respectively (Figure 1E), indicating that both constructs effectively induce endogenous gene editing.

The editing profiles were further analysed by sequencing. Eighteen lines (81.8%) contained putative homozygote/biallelic mutations in all subgenomes at the sgRNA1 target site. In some lines, putative homozygote/biallelic mutations exist in one subgenome, while heterozygote or chimeric mutations appear in other subgenomes (T0‐10 and T0‐26; Figure 1F). Eight mutation types were identified from 590 independent clones (Figure 1G). The most frequent mutation type was deletion (75%), of which 59.1% are small deletions (<2 bp). The ratios of large fragment deletions (≥14 bp), insertions and combined indels were 15.9%, 2.21% and 7.82%, respectively (Figure 1G). Since bamboo propagates through asexual budding, those homozygote/biallelic mutations will remain in the genome of their offspring clones during breeding.

sgRNA2 that perfectly targets *DlmPSY1‐A1,* but not *DlmPSY1‐B1* or *DlmPSY1‐C1* was designed to study the recognition specificity (Figure 1D). Sequencing results confirmed that 10 transgenic lines contain mutations in *DlmPSY1‐A1*, but none in *DlmPSY1‐B1* and *DlmPSY1‐C1* (Figure 1E). Two lines (20%) were putative homozygous or biallelic mutations (T0‐12 and T0‐14), while 7 lines (70%) were heterozygous/chimeric (T0‐30 to T0‐32 as representative examples, Figure 1H). The ratios of deletions, insertions and combined mutations were 86%, 9% and 5%, respectively (Figure 1I). The mutations were predominantly short nucleotide changes (1–26 bp), and 22.7% were 1bp nucleotide deletions (Figure 1I). Those data demonstrated the successful application of the CRISPR/Cas9 system in mutating a specific *DlmPSY1* allele.

Eighteen lines (81.8%) with homozygote/biallelic mutations in all subgenomes at the sgRNA1 site exhibited albino phenotypes (Figure 1J), which appeared at an early stage during tissue culture and persisted at the plantlets stage (Figure 1J). Those results suggest that genome editing takes place at an early stage in embryonic cells and led to the loss of function of all *DlmPSY1* alleles. Similar results were reported in rice, wheat or cotton (Wang *et al.*, 2014; Wang *et al.*, 2018; Zhang *et al.*, 2014). In case of sgRNA2, although *DlmPSY1*‐*A* was mutated, no visible phenotypic change was observed due to the existence of the wild‐type *DlmPSY1‐B* and *DlmPSY1‐C* alleles.

Next, we applied this technology in bamboo molecular research. Bamboo is the tallest grass in the world, while the underlying mechanism is unknown. Previously, we identified several Gibberellin‐responsive genes including *GRG1* (GA‐responsive gene 1, *PH01004823G0070*) that potentially acts in controlling bamboo height (Zhang *et al.*, 2018). Here, two homozygote *grg1* mutants (efficiency 40%) in Ma bamboo were produced using our optimized CRISPR/Cas9 technology. Mutation in *GRG1* increased plant height **(**Figure 1K), mostly due to elongated internodes (Figure 1L‐N). Sequencing results confirmed that the *grg1* mutant has the putative homozygous mutation in A1 subgenome, biallelic mutation in B1 subgenome and homozygous mutation in C1 subgenome (Figure 1O), indicating the loss of function of *GRG1* in transgenic bamboo. To our knowledge, this is the first example on controlling bamboo height through gene manipulation, which will contribute to subsequent studies on the molecular mechanisms behind the fast growth of bamboo.

In summary, for the first time we engineered the hexaploid Ma bamboo through CRISPR/Cas9 technology. The homozygote mutations were obtained in the first generation of transgenic lines, which are extremely important for bamboo species due to its long vegetative growth periods. We also confirmed the albino phenotype of *dlmpsy1* mutant in bamboo and generated a bamboo mutant with altered plant height. This demonstrates the applicability of CRISPR/Cas9 in bamboo and thereby boosts future bamboo research and breeding.

## Funding

This work was supported by the National Natural Science Foundation of China grant (No. 31870660), Fujian Innovative Center for Germplasm Resources and Cultivation of Woody plants (No. 125/KLA15001E), Program for scientific and technological innovation team in university of Fujian province (No. 118/KLA18069A) to Q.Z. The funding bodies were not involved in the design of the study or in any aspect of the data collection, analysis and interpretation of data and in paper writing.

## Conflict of interests

The authors declare that they have no conflict of interests.

## Authors’ contributions

Q.Z. conceived this project. L.F.G., Y.S.Z., X.Q.M. C.T.L. and Q.Z. designed experiments and interpreted the results. S.W.Y., G.C. and M.V.K. performed the experiments and analysed the data. W.J.W., C.Y.C., C.W. and D.W.S. helped to perform the experiments and collect the data. All authors read and approved the submission of this manuscript.
